# Mitochondrial DNA lineages of African origin confer susceptibility to primary open-angle glaucoma in Saudi patients

**Published:** 2011-06-03

**Authors:** Khaled K. Abu-Amero, Ana M. González, Essam A. Osman, José M. Larruga, Vicente M. Cabrera, Saleh A. Al-Obeidan

**Affiliations:** 1 Department of Ophthalmology, College of Medicine, King Saud University, Riyadh, Saudi Arabia; 2 Department of Genetics, Faculty of Biology, University of La Laguna, Tenerife, Spain; 3 Department of Ophthalmology, College of Medicine, University of Florida, Jacksonville, FL

## Abstract

**Purpose:**

We previously reported that certain mitochondrial DNA (mtDNA) polymorphisms in the coding region may be involved in the pathogenesis for primary open-angle-glaucoma (POAG). This encouraged us to extend our work and assess whether mtDNA diagnostic polymorphisms, defining geographically structured haplogroups, could be associated with the development of POAG.

**Methods:**

We sequenced the mtDNA regulatory hypervariable region-I (HVS-I) region and coding regions, comprising haplogroup diagnostic polymorphisms, in 176 POAG patients and 186 matched healthy controls (free of glaucoma by examination) of Saudi Arabia ascendancy. A large sample of 810 healthy Saudi Arabs representing the general Saudi population has also been included in the analysis. Assigning individuals into various mitochondrial haplogroups was performed using the nomenclature previously described for African and for Eurasian sequences.

**Results:**

African mtDNA haplotypes belonging to L haplogroups, excluding L2, confer susceptibility to POAG whereas the Eurasian haplogroup N1 was associated with reduced risk of developing POAG in Saudi Arabian population.

**Conclusions:**

Saudi individuals with mtDNA of African origin are at higher risk of developing POAG. In addition, the mtDNA Eurasian haplogroup N1 may play a mild protective effect to this illness.

## Introduction

The term glaucoma comprises a heterogenous group of ocular disorders characterized by progressive retinal ganglion cell death, optic nerve atrophy and different patterns of visual field loss. There are two types of primary glaucoma: Primary open-angle-glaucoma (POAG), and primary angle-closure glaucoma (PACG). POAG is the most common type worldwide accounting for majority of glaucoma cases [1]. Older age, positive family history, age, black race, myopia, diabetes, hypertension, and elevated ocular pressure have been identified as important risk factors for POAG. The prevalence of POAG in Saudi Arabia is unknown. The Glaucoma unit at King Abdulaziz University Hospital (KAUH), where approximately 600 new glaucoma patients are seen annually (as indicated by an ongoing study on the pattern of glaucoma at KAUH for the period from 2006 to 2010), has found that 19% of those are POAG, 40% primary angle*-*closure glaucoma, 10% have pseudoexfoliation glaucoma, and the remaining 31% are other types of glaucoma. It seems that racial and sex influences depend on the type of glaucoma. POAG has been reported with higher prevalence in African and men [2] and PACG in Asians and women [3]. Primary glaucoma is also heterogeneous in its hereditable basis. Although rare forms of juvenile glaucoma may be caused by single gene mutations, the majority of cases are better explained by a confluence of complex genotype and environmental risk factors [4,5]. It has also been suggested that, similar to other optic nerve atrophies, mitochondrial dysfunction or altered mitochondrial signaling pathways are involved in the glaucoma pathogenesis [6,7]. In a recent article on POAG in Saudi Arabia [8], no pathological nucleotide changes were detected for the autosomic causative genes myocilin (*MYOC*) and optineurin (*OPTN*). Conversely, the number of nonsynonymous mutations and transversions found in the mitochondrial DNA (mtDNA) coding region of POAG patients was significantly greater than in control subjects. In addition, mean mitochondrial respiratory activity was decreased by 21% in POAG patients compared with control subjects. Although no individual mutations could be associated with POAG, these data suggested that mitochondrial dysfunction may be a risk factor for this disease and were in accordance with previous epidemiological studies showing that the prevalence of a positive maternal family history of POAG patients was significantly greater than on the paternal side [9]. Due to its maternal inheritance, high mutation rate and lack of recombination mtDNA shows a distinct picture of female human dispersion, resulting in the distribution of its lineages into continent specific haplogroup. These haplogroups are defined by diagnostic polymorphisms present in the mtDNA coding and regulatory regions. Thus, at the broadest level, mtDNA sequences belonging to macro-haplogroup L had an African origin and those belonging to macro-haplogroups M and N had a Eurasian origin. There are numerous reports associating mtDNA haplogroups to various diseases including optic neuropathies [10]. The aim of the present study was to assess the possible role of mtDNA haplogroups in POAG among Saudi patients.

## Methods

### Patients and control subjects

We recruited 176 Saudi POAG patients who satisfied strict clinical criteria for POAG which includes the following: i) appearance of the disc or retinal nerve fiber layer e.g., thinning or notching of disc rim, progressive changes, nerve fiber layer defect; ii) the presence of characteristic abnormalities in visual field (e.g., arcuate scotoma, nasal step, paracentral scotoma, generalized depression) in the absence of other causes or explanation; iii) age greater than 40 years, and iv) open anterior chamber angles bilaterally on gonioscopy. Exclusion criteria included evidence of secondary glaucoma, e.g., pigmentary dispersion syndrome, pseudoexfoliation, history of steroid use, or ocular trauma. All cases had onset of glaucoma after age 40 (adult-onset POAG). Patients were recruited from the glaucoma clinic at KAUH after signing an informed consent approved by the institutional review board (proposal number # 08–657).

A second group (n=186) of healthy Saudi Arabs controls (HMC group) free from glaucoma by examination were recruited. Entry criteria for those subjects were age >40, normal IOP, open angles on gonioscopy, and normal optic nerves upon examination.

A third large sample (n=810) of healthy Saudi Arabs (HSA), representing all five major Saudi Arabian provinces, that were recruited previously for population genetics studies, was used for statistical comparisons. All individuals of this group were Saudi Arabs who reported no symptomatic metabolic, genetic, ocular disorders or any ophthalmic problem on an extensive questionnaire regarding family history, past medical problems, and current health. For those controls, their mother’s ancestral origin was established as a Saudi. The mtDNA haplogroup assignation for 552 subjects of this sample has been already published [11]. All patients and controls were maternally unrelated Saudi Arabs, all whose known ancestors were of Saudi Arabian origin. This research followed the tenets of the Declaration of Helsinki.

### DNA extraction

Five milliters of peripheral blood were collected in EDTA tubes from all participating individuals. DNA was extracted using the illustra blood genomicPrep Mini Spin Kit from GE Healthcare (Buckinhamshire, UK), and stored at –20 °C in aliquots until required.

### Mitochondrial haplogroup assortment

All samples were amplified and sequenced for the mtDNA regulatory region hypervariable segments (HVSs) I and II using previously described primers and conditions [12]. Haplotypes were tentatively assorted into haplogroups according to their HVS diagnostic positions. This assortment was further confirmed, when necessary, by the analysis of coding-region diagnostic haplogroup polymorphisms (Table 1), following the most recent mtDNA haplogroup nomenclature [13]. To detect these polymorphisms a fragment spanning the diagnostic position was amplified and sequenced using any of the 32 overlapping pairs of primers that cover the whole mtDNA genome, and the PCR and sequencing conditions previously published for each of them [12].

**Table 1:** mtDNA haplogroup distribution in POAG patients and controls.

**Haplogroup**	**Coding positions**	**POAG (n=176)**	**HMS (n=186)**	**HAS (n=810)**	POAG* HMS p value	POAG* HSA p value
H	C7028C	13 (7.4%)	9 (4.8%)	66 (8.1%)	0.31	0.74
R0a	T3847C	34 (19.3%)	26 (14.0%)	141 (17.4%)	0.17	0.55
J	T4216C, C15452A	37 (21.0%)	47 (25.3%)	171 (21.1%)	0.34	0.98
T	T4216C, G15928A	6 (3.4%)	9 (4.8%)	53 (6.5%)	0.50	0.11
K	A3480G	3 (1.7%)	4 (2.2%)	27 (3.3%)	0.76	0.25
U	A12308G, 12372A	16 (9.1%)	25 (13.4%)	91 (11.2%)	0.19	0.41
Other R	C12705C	3 (1.7%)	8 (4.3%)	25 (3.1%)	0.15	0.32
N1	T10238C, 12501A	8 (4.5%)	13 (7.0%)	66 (8.1%)	0.25	0.04*
W	G15884C	0	0	6 (0.7%)	-	0.25
X	C6371T	2 (1.1%)	4 (2.2%)	20 (2.5%)	0.45	0.28
M1	C10400T, 12403T	10 (5.7%)	11 (5.9%)	25 (3.1%)	0.93	0.09
M(xM1)1	C10400T	6 (3.4%)	6 (3.2%)	24 (3.0%)	0.92	0.76
L	T9540C, 10400C	38 (21.6%)	24 (12.9%)	95 (11.7%)	0.03*	0.001***
L2	T10115C	11 (6.3%)	10 (5.4%)	35 (4.3%)	0.72	0.27
L(xL2)2	T9540C, 10400C	27 (15.3%)	14 (7.5%)	60 (7.4%)	0.02*	0.001***

POAG, Primary open-angle glaucoma patients; HMC, healthy matched controls; HAS, healthy Saudi Arab sample. χ^2^ or Fisher exact tests were applied to investigate the association between having a certain haplogroup and the occurrence of POAG. 1. All M haplogroups excepting M1. 2. All L haplogroups excepting L2.

### Statistical analysis

Haplogroup frequency differences between patient and control cohorts were tested by pair-wise-exact tests of sample differentiation [14] using the Arlequin 3.11 package. The frequency of determined haplogroups, between cases and controls were compared with the ÷^2^ test or with Fisher’s exact tests when appropriate. To keep statistically adequate haplogroup sub-sample sizes within cohorts, when necessary, mtDNA haplogroups were collapsed into larger haplogroup identities following a phylogenetic criterion [13].

## Results

Haplogroup frequencies obtained for the POAG patients, healthy matched controls (HMC) and the general Saudi Arab population (HSA) are listed in Table 1. Pair-wise exact tests between cohorts, based on total haplogroup frequencies, showed that HMC and HSA controls are homogenous groups (p=0.516). On the other hand, the POAG cohort differs significantly (p=0.028) from HSA controls only. A haplogroup by haplogroup inspection (Table 1) shows that haplogroup L frequency (0.216) and, in less degree, to the N1 (0.045) frequency in POAG as the most different from HSA controls (0.117 and 0.081, respectively). In fact, ÷^2^ test analysis confirmed that haplogroup L was in a significant excess (p=0.001) and haplogroup N1 in a significant decrease (p=0.04) in POAG compared to the HSA group. Furthermore, matched controls HMC also presents a significant decrease of L haplotypes (0.129) compared to POAG (p=0.03) and have, comparatively, more N1 haplotypes (0.07) although without reaching significance (p=0.250). These data could be interpreted as that, the African haplogroup L is associated with the risk of developing POAG while the Eurasian haplogroup N1 could have a mild protective effect on this illness.

## Discussion

From the results obtained here, it can be deduced that there is a POAG predisposition in those Saudi individuals with mtDNA of African ancestry. The significantly greater susceptibility to POAG in subjects with African ascendance compared to those with Eurasian ascendance, in other geographical areas, has been well documented [2,15]. Our results are congruent with the lack of differences in the haplogroup distribution between POAG patients and healthy controls in a study performed on white people from England [16] as they did not analyzed the sub-Saharan African segment of the English population. They could also satisfactorily explain the higher number of mtDNA non-synonymous mutations found in POAG Saudi patients when compared with healthy individuals found in a previous mtDNA genomic analysis [8]. All the sequences are compared to the revised Cambridge reference sequence (rCRS) [17], that is an Eurasian sequence and, therefore, more phylogenetically related to other Eurasian sequences than to the L African sequences [13]. Now, we know that the frequency of African haplogroups in POAG is significantly greater than in controls so, it is logical that a higher number of mutations were found in the POAG group compared to the control group. Furthermore, several of the mtDNA coding region mutations found in Saudi POAG patients in that study, have been found in Sub-Saharan African complete sequences. These results illustrate the convenience of knowing the haplogroup background of individual mitochondrial variants with significant association to diseases because, as in our case, it could unveil unknown racial risk factors for those diseases or unexpected population substructure. It is also well known that other ethnic groups are more propense to other types of glaucoma. In the case of the Saudi Arabian population, it has been recently found that patients with haplotypes belonging to the mtDNA haplogroup R0a are at higher risk of developing PACG [18]. As R0a is a Eurasian haplogroup, this result is also in agreement with the higher predisposition found for PACG in Asians. Both results confirm, in Saudi Arabia, that multi-ethnic populations have different human groups with different illness predispositions. Even when there are not behavioral barriers among groups, as the mtDNA is a non-recombining molecule, the original ethnic maternal ancestry of an individual can be easily detected and its influence evaluated independently of its nuclear genome background. In our samples, the mtDNA variant 16189T>C has a frequency significantly greater (p<0.000) in African haplogroups (0.38) than in Eurasian haplogroups (0.18). Frequently, this polymorphism has been found associated to different diseases, including coronary artery disease and myocardial infarction in Saudi Arabs [19], but it seems not to play a particular role in POAG. First, the frequency differences of 16189 between POAG and control cohorts HMC or HSA did not reach significance (p=0.24 and p=0.12, respectively). Second, within African haplogroups, the frequency of 16189 in POAG (0.39) is strikingly similar to that in HNC (0.39) or HSA (0.37) and the same happens when its frequency, within Eurasian haplogroups, is compared between the same cohorts (0.21, 0.17, and 0.17, respectively). In a parallel association study involving mtDNA and pseudoexfoliation glaucoma (PEG) in Saudi Arabia [20], we found that the African mtDNA haplogroup L2 and the Eurasian haplogroup T confer susceptibility to PEG whereas the Eurasian haplogroup N1 was associated with reduced risk to develop that illness. At first sight, these results are strikingly similar to those found in the present study: the African macro-haplogroup L and the Eurasian N1 were found respectively associated with susceptibility and reduced risk to POAG. On its side, haplogroup T has also a lower frequency in POAG (0.03) than in both HMC (0.05) and HSA (0.07) controls, although differences did not reach a significant level (p=0.50 and p=0.11, respectively). Moreover, these analogous mtDNA associations in POAG and PEG coincide with another recent study reporting that mitochondrial damage in the trabecular meshwork occurs only in these two forms of glaucoma [7]. However, the association found between PEG and L haplotypes was specifically with those belonging to haplogroup L2, whereas the one found here involves different L haplotypes as there is a lack of specific association between POAG and haplogroup L2 when compared to HMC (p=0.72) and HAS (p=0.27) controls. Differences in co-adaptation between mitochondrial and nuclear genomes might be responsible of this difference. There is increasing information about the role of mitochondria in the development of POAG [6,21]. It seems that mtDNA polymorphisms, defining haplogroups are also involved in this process. However, to unveil the genetic and physiologic causes of these associations further research is needed.
